# Efficient and heritable transformation of *Phalaenopsis* orchids

**DOI:** 10.1186/s40529-016-0146-6

**Published:** 2016-10-20

**Authors:** Hong-Xian Hsing, Yi-Jyun Lin, Chii-Gong Tong, Min-Jeng Li, Yun-Jin Chen, Swee-Suak Ko

**Affiliations:** 1 Academia Sinica Biotechnology Center in Southern Taiwan, Tainan, 741 Taiwan; 2 Agricultural Biotechnology Research Center, Academia Sinica, Taipei, 115 Taiwan

**Keywords:** *Phalaenopsis aphrodite*, *Agrobacterium tumefaciens*, Transformation, Protocorm, eGFP, Hygromycin selection

## Abstract

**Background:**

*Phalaenopsis* orchid (*Phal.* orchid) is visually attractive and it is important economic floriculture species. *Phal.* orchids have many unique biological features. However, investigation of these features and validation on their biological functions are limited due to the lack of an efficient transformation method.

**Results:**

We developed a heritable and efficient *Agrobacterium*- mediated transformation using protocorms derived from tetraploid or diploid *Phal.* orchids. A T-DNA vector construct containing eGFP driven by ubiquitin promoter was subjected to transformation. An approximate 1.2–5.2 % transformation rate was achieved. Genomic PCR confirmed that hygromycin selection marker, *HptII* gene and target gene *eGFP* were integrated into the orchid genome. Southern blotting indicated a low T-DNA insertion number in the orchid genome of the transformants. Western blot confirmed the expression of eGFP protein in the transgenic orchids. Furthermore, the GFP signal was detected in the transgenic orchids under microscopy. After backcrossing the pollinia of the transgenic plants to four different *Phal.* orchid varieties, the BC1 progenies showed hygromycin resistance and all surviving BC1 seedlings were *HptII* positive in PCR and expressed GFP protein as shown by western blot.

**Conclusions:**

This study demonstrated a stable transformation system was generated for *Phal.* orchids. This useful transformation protocol enables functional genomics studies and molecular breeding.

**Electronic supplementary material:**

The online version of this article (doi:10.1186/s40529-016-0146-6) contains supplementary material, which is available to authorized users.

## Background

*Phalaenopsis* orchids, commonly known as moth orchid, is one of the most popular and profitable ornamental plants in the global floral market. *Phal.* orchids have very unique biological features such as crassulacean acid metabolism (CAM) photosynthesis that uptake CO_2_ at night (Guo and Lee 2006), epiphytic habitat with high water and nutrient usage efficiency, unique flower pattern formation (Su et al. 2013b), symbiosis with mycorrhizae (Yumiko et al. 2007), immature embryos (seeds without endosperm) in mature capsules (Yu and Goh 2001). To elucidate these interesting and novel biological features, validation of gene function using stable transformation approach must be helpful. Moreover, the recent technological advances in next generation sequencing (NGS) combining bioinformatics have generated highly abundant sequence information. Several orchid databases based on the transcriptomic information are constructed and available in the web, such as Orchidstra (Su et al. 2013a) and OrchidBase (Fu et al. 2011). In the era of big data and functional genomics, efficient transformation of *Phal.* orchid can contribute to the new discoveries with strong impact.

Over the years consumers have sought ever more attractive and versatile morphological traits in flowers, such as colors, shapes, fragrance. Although conventional breeding has been fruitful in last decades, to meet the demands of the market for novel floral colors or fragrances, control of flowering time, resistant to diseases and environmental stresses and so on, transgenic technology is needed. Introgression of foreign genes from distant species may create more dramatic outcomes in transgenic lines than conventional breeding.

According to previous studies, the most reliable genetic transformation platforms successfully applied to *Phal.* orchids are the *Agrobacterium*-mediated approach and particle bombardment (Chai et al. 2002; Mii and Chin 2010; Teixeira da Silva 2011). Target explants for transformation using protocorms (Chin et al. 2007; Mishiba et al. 2005; Semiarti et al. 2007, 2010), protocorm-like bodies (PLB) (Anzai et al. 1996; Anzai and Tanaka 2001; Chai et al. 2002; Chan and Lin 2005; Chen et al. 2011; Hsieh et al. 1997; Julkifle et al. 2010; Liao et al. 2004; Subramaniam et al. 2009; Subramaniam and Xavier 2010), callus (Belarmino and Mii 2000; Sjahril and Mii 2006); and pollen tube pathways (Hsieh and Huang 1995; Tsay et al. 2012) have been reported. So far, the two most-often used host orchid tissues, protocorms and PLBs have been genetically transformed equally well. PLBs are presumed to be genetically uniform and can be induced efficiently from various somatic tissues including flower stalks, young leaves and stem segments (Chen et al. 2010; Yee et al. 2008). However, protocorm transformations have been proposed to be simpler than subculture of PLBs (Mishiba et al. 2005; Semiarti et al. 2007).

In the present study, we developed an alternative transformation procedure using protocorms generated by germination of seeds derived from diploid *Phal. aphrodite* and tetraploid *Phal.* cultivars. A T-DNA vector construct containing eGFP driven by ubiquitin promoter was used for transformation. The transformed protocorms were selected by hygromycin and subsequently regenerated successfully. We backcrossed the transgenic line and the BC1 progenies showed hygromycin resistant and the transgene is heritable. Finally, molecular analyses using PCR and western blot showed that all surviving backcross F1 explants were positive transformants.

## Methods

### Plant materials and growth condition

**Fig. 1:**
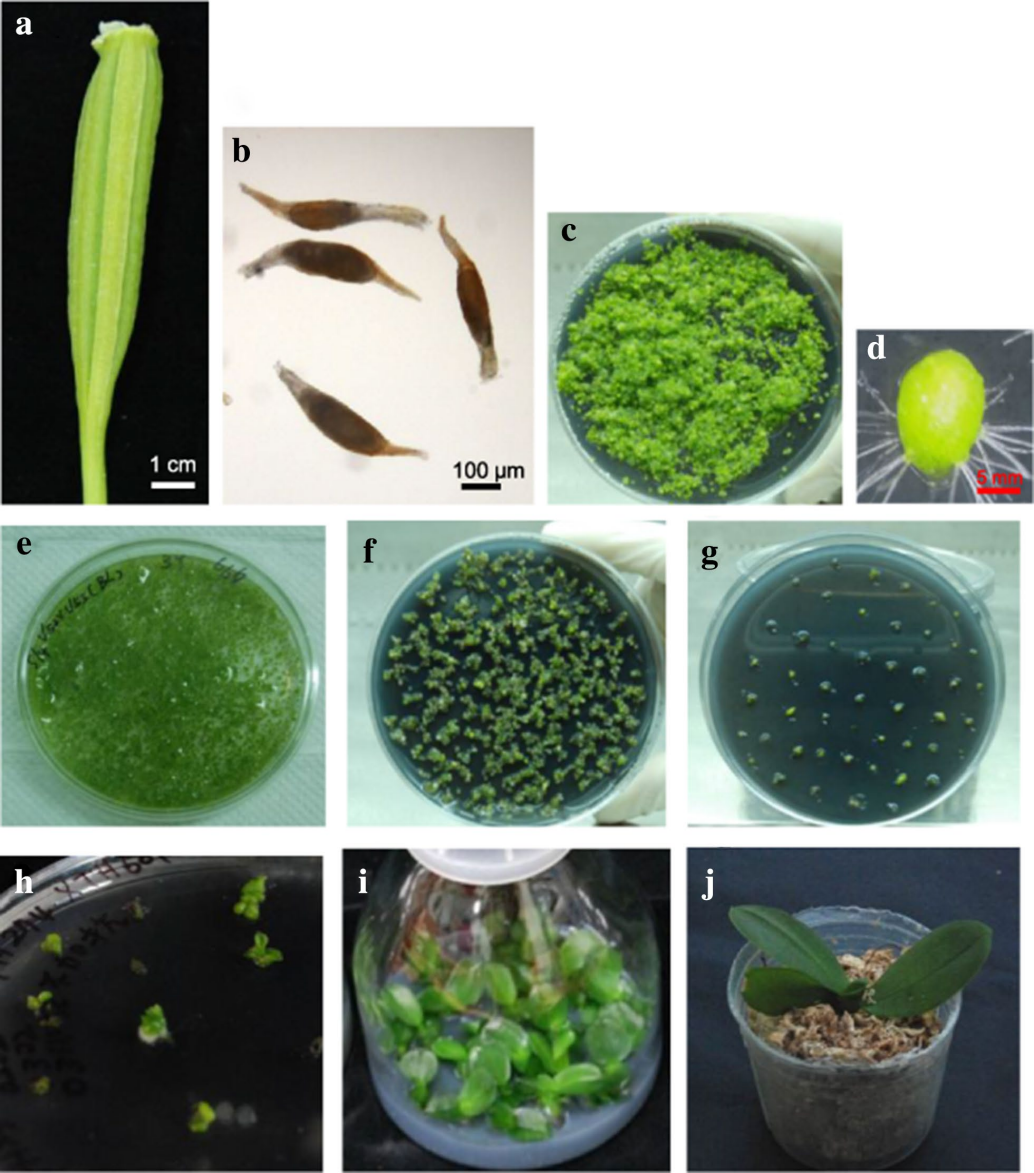
Procedures for *Agrobaterium*-mediated transformation of protocorms in *Phalaenopsis* orchid. **a** Mature orchid capsule at 4 months after pollination. **b** Stage of mature seeds ready for sowing. **c** Growth of protocorms sown on 1/2 MS agar plates for 1 month. **d** Healthy protocorm ready for infection. **e** *Agrobacteria* infection. **f** The 1st round selection for hygromycin resistance on T2 media containing 25 ppm hygromycin and 40 ppm meropenem (T2 + MH). **g** The 2nd round selection for hygromycin resistance on T2 + MH. **h** Growth of explants on 1/2 MS plate. **i** Growth of transgenic lines in jar. **j** Transplanted transgenic orchid to 1.7″ pot containing sphagnum moss

Three different *Phalaenopsis* orchid varieties were used for *Agrobacterium*- mediated transformation in this study. A popular big white flower tetraploid orchid of Dpts. Join Angel ‘TH274-1’ was bought from Join Orchids. It was self-pollinated and single seed descendant line (denoted L3) was used in our transformation. A commercial tetraploid orchid of *Phal. aphrodite* cv. M1663 (denoted Ama 4n) was bought from Chain Port Orchid Co. Ltd. A native Ama diploid of *Phal. aphrodite* subsp. *formosana* (denoted Ama 2n) was bought from the market of Shanhua. Plant-type and flower morphology of the three *Phalaenopsis* orchids is shown in the Additional file 1: Figure S1. *Phal.* orchid L3 with large white flowers more than 10 cm in diameter; whilst Ama (4n) and Ama (2n) have medium size white flowers around 7 cm in diameter. When blooming, flowers were self-pollinated to produce capsules. It took about 4–5 months for capsules to mature. Capsules were harvested when they turned slightly yellowish green at the tip of the capsule (Fig. 1a). Mature capsules could be stored at room temperature or 4 °C for 2 weeks and seeds still maintained good viability. Orchid seeds were sown on a 1/2 MS (Duchefa Bi ochemia) agar plate containing 0.1 % activated charcoal and placed in a tissue culture room at constant temperature of 24 °C under a 12 h photoperiod.

### Plasmid vectors and bacterial strain

**Fig. 2:**
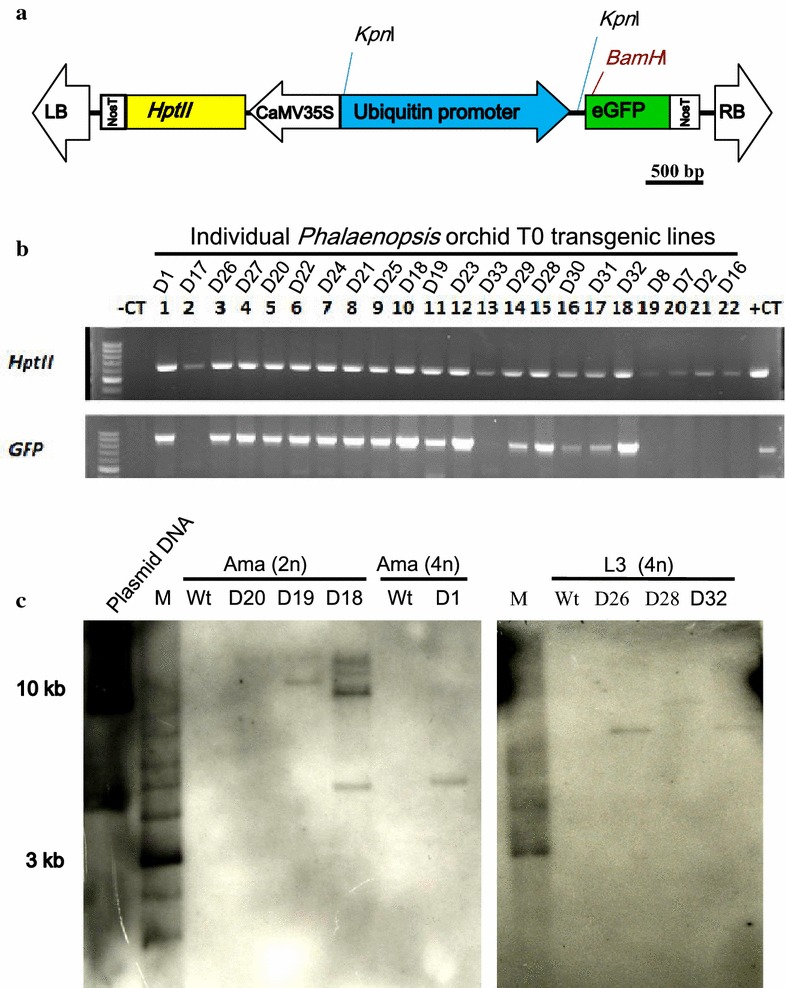
Confirmation of *Phalaenopsis* transgenic lines. **a** Construction map of Ubi:eGFP used in this study. Schematic structure of the T-DNA region in the vector of pZp200. CaMV 35S, promoter of CaMV 35S; *HptII*, coding region of *hygromycin phosphotransferase* gene; eGFP, coding region of *eGFP*; NosT, terminator of *nopaline synthase* gene; *LB* left border; *RB* right border. **b** Genomic PCR analysis of *eGFP* and *HptII* genes. −CT, negative control without addition of the genomic DNA template; +CT, with added plasmid DNA. **c** Southern blot analysis of *HptII* showing integration of T-DNA in *Phalaenopsis* orchid genome. Transgenic lines and their respective Wt plants were analyzed side by side

A green fluorescence marker gene eGFP driven by ubiquitin promoter was constructed into pZP200 backbone (http://www.biotech.unl.edu/pzp200) containing the selection marker *HptII* gene that encodes *hygromycin phosphotransferase,* was driven by cauliflower mosaic virus (CaMV) 35S promoter (Fig. 2a). The vector, pCAMBIA1305.1, which contained a β-glucuronidase (*GUS*) was driven by CaMV 35S promoter (http://www.cambia.org/daisy/cambia/585.html). The plasmid was separately subjected to transformation and subsequent hygromycin selections. *Agrobacterium tumefaciens* strain EHA105 was used for transfection in this study.

### Inoculation, co-cultivation, and selection

*Agrobacterium* strain EHA105 containing eGFP target gene was spread on lysogeny broth (LB) plate containing 50 ppm kanamycin and 100 ppm rifampicin, and cultured overnight. Single colonies were selected and then cultured in 5 mL LB solution containing 50 ppm kanamycin and 100 ppm rifampicin, shaking at 250 rpm in a 28 °C incubator for 2 days. Agrobacteria were transferred to 20 mL LB containing 50 ppm kanamycin and 100 ppm rifampicin, and incubated at 28 °C to reach an optical density (OD) of a suspension of cells at 600 nm of around 1.0. Inoculum was centrifuged at 3500 rpm for 15 min, then the pellet was re-suspended with 1/2 MS containing 5 % sucrose, adjusted to pH 5.7, then 100 μM acetosyringone (AS) was added to the inoculum.

Healthy *Phal.* protocorm of size ~1.2 mm were selected, placed on a petri dish, and immersed in the *Agrobacterium* inoculum for 25 min. *Agrobacterium* inoculum was removed and the infected protocorms were spread on New Dogashima medium (NDM) (Belarmino and Mii 2000) containing 10 g/l maltose, 0.1 ppm naphthaleneacetic acid, 0.4 ppm benzyladenine (BA), 100 μM acetosyringone (AS), and 0.29 % phytagel. Infected protocorms were co-cultivated in the dark for 2–3 days until growth of *A*. *tumefaciens* was observed. Protocorms were washed with distilled water containing 40 ppm meropenem (China Chemical & Pharmaceutical) 3–4 times, and afterwards the protocorms were spread on T2 plates (Chen et al. 2009) containing 25 ppm hygromycin and 40 ppm meropenem (T2 + MH). Antibiotic selection of putative transgenic explants was conducted on T2 + MH three times, at 2-week intervals.

### Transformation rate

To test the amenability of this protocol, a batch of experiments was carried out in triplicate using Ubi:eGFP construct. The numbers of the initially germinated protocorms of TH274 × TH601 hybrid and surviving protocorms that were resistant to hygromycin from the first to the third round of selection were recorded. Parental lines of TH274 and TH601 were purchased from Join Orchids. The percentage transformation rate was calculated based on the explants surviving after the third round of selection.

### DNA isolation

Genomic DNA was isolated from a small piece of leaf tissue about 2 × 1 cm taken from putative transgenic lines using the cetyltrimethylammonium bromide (CTAB) method (Lee et al. 2015). The genomic DNA was used for PCR as well as for Southern blot analysis.

### PCR determined transgenic lines

Polymerase chain reaction (PCR) was used to determine transgenic lines using *HptII* primers 5′-GATGTAGGAGGGCGTGGATA-3′ and 5′-CGTCTGCTGC TCCATACAAG-3′ and the target gene was confirmed by using primers eGFP 5′-ATGGTGAGCAAGGGCGAGGA-3′ and Nos-R 5′-ATCGCAAGACCGGCAACAGGA-3′. The amplicon sizes are predicted to be 621 and 930 bp, respectively. The PCR amplification was carried out using following conditions: 95 °C, 3 min (1 cycle), 95 °C, 1 min, 58 °C, 1 min, 72 °C, 1 min (34 cycles), 72 °C, 10 min. Annealing temperature for *HptII* and *eGFP* was 58 and 60 °C, respectively.

For early detection of gene insertion, a small portion of leaf tissue (1 × 1 mm) was excised from small seedlings after 2 months recovery from the 3rd round hygromycin selection. DNA was directly extracted using Phire Plant Direct PCR Kit (Thermo Scientific) and PCR was performed using the *HptII* primers.

### Southern blot analysis

Genomic DNA was extracted from leaf tissues (1 g fresh weight) using the CTAB method. After quantification by gel electrophoresis, 10 μg of each DNA was digested with *BamHI* and fractionated on a 0.8 % agarose gel. Southern hybridization and detection were carried out using a digoxigenin-labeled *HptII* probe following the manufacturer’s instructions (Roche, http://www.rocheapplied-science.com).

### Western blot analysis

Total protein of leaf samples was extracted with Culture Cell Lysis Reagent (CCLR) buffer (100 mM K_2_HPO_4_, 100 mM KH_2_PO_4_, pH 7.8, containing 1 % Triton X-100, 10 % glycerol, 1 mM EDTA, and 7 mM 2-mercaptoethanol). The protein amount was measured by using the Bio-Rad Protein Assay Kit with bovine serum albumin as a standard. For western blot analysis, 30 µg total protein from each sample was loaded and separated by SDS-PAGE with a 10 % acrylamide gel and transferred onto polyvinylidene fluoride (PVDF) membrane for antibody probing. The following primary antibodies were used: anti-eGFP, rabbit polyclonal antibody (Yao-Hong Biotechnology, Cat#YH-80005) at 1:10,000 dilution and anti-Actin mouse monoclonal antibody (Sigma, A0480) at 1:2500 dilution. Horseradish peroxidase (HRP)-conjugated secondary antibodies were obtained from GeneTex. Secondary anti-rabbit antibody was diluted at 1:10,000; anti-mouse antibody was diluted at 1:12,000.

### GFP fluorescence microscopy

Leaf and root tissues of the D20 transgenic line and the non-transgenic wild-type were used for GFP florescence observation. Root tissue excised about 8 mm from the root tips and leaves was sampled from the mid vein of young developing leaves. Tissues were embedded in 4 % agarose II low gelling temperature (AMERESCo, Lot# 1624co42). The agarose sample block was trimmed to a desired size and adhered to a specimen stage. Vertical and transverse sections of the roots were made, and leaves were transverse sectioned. Tissues were sectioned to ~120 µm thickness using a blade installed in the Vibratome 1000 plus sectioning system (Fedelco, SL). A GFP signal was detected and an image was taken using a Zeiss LSM710 confocal microscope equipped with a T-PMT under an FITC filter at excitation of 488 nm and emission wavelength of 500–560 nm.

### GUS assay

T0 explants of 35S:GUS and non-transgenic wild type were stained with X-Gluc and incubated at 37 °C overnight according to a previously described protocol (Jefferson et al. 1987). Chlorophyll was removed by immersing in 95 % ethanol for 24 h. Pictures of whole explants were taken using a Nikon SMZ1500 dissecting microscope at 7.5× magnification. For detailed observation of the GUS expression pattern in transgenic cells, explants were sectioned using a vibratome according to the protocol mentioned in the previous section.

### Inheritance of transgene

Transgenic orchid was crossed to non-transgenic wild types to generate BC1 segregating populations to determine if the transgene is heritable. One T0 plant (line D1) bloomed giving two flowers. A total of four D1 pollinia were backcrossed to four independent female parental non-transgenic *Phal.* orchids in a distinct genetic background, i.e., Ama (4n, the original Wt of D1), Ama (2n), C2-4 hybrid (TH274 × *Phalaenopsis equestris*), and V65 × V15 (hybrid orchid), respectively. Parental lines of *Phalaenopsis equestris*, V65 and V15 were purchased from Jumbo Orchids, Kaohsiung Guanyinshan and Chain Port Orchid Co. Ltd., respectively. Approximately four months after pollination, orchid capsules were harvested and the BC1 seeds were sown on a 1/2 MS plate containing 25 ppm hygromycin. Two months after sowing, the numbers of explants that were resistant or susceptible to hygromycin were calculated. The surviving green seedlings were considered to be transgenic lines that were resistant to hygromycin, whilst those wilting or undeveloped protocorms were hygromycin susceptible wild-type like progenies. In order to confirm introgression of the transgene to the next generation, 22 BC1 s of Ama (4n)/D1, Ama (4n) Wt as a negative control and D1 (the original T0 transgenic plant) as a positive control were applied to PCR detection using *HptII* primers and western blot using eGFP antibody.

## Results

### Transformation processes and obtained transgenic seedlings

Mature orchid capsules were harvested (Fig. 1a) and surface sterilized with 70 % EtOH. The capsule was cut longitudinally with a sterilized scalpel and tiny seeds were spread evenly on 1/2 Murashige and Skoog (MS) agar plates. Seed germination and growth of protocorms took place on agar plates at constant temperature of 24 °C (Fig. 1b). Five weeks after sowing, protocorms had grown substantially and had turned light green in color (Fig. 1c). Healthy globular shape protocorms of approximately 1.2 mm in length (Fig. 1d) were selected and infected with *Agrobacterium* inoculum (Fig. 1e). Selection of putative transgenic lines was performed by three successive hygromycin selections at 2-week intervals per selection (Fig. 1f–h). The greenish explants after the 3rd round of antibiotic selection (Fig. 1h) were recovered in a 1/2 MS agar plate for 2 months and then transferred to a jar for approximately 3 months before transplanting the transgenic orchid to a 1.7” pot containing sphagnum moss. Finally, a total of 74 independent transgenic orchid seedlings overexpressing Ubi:eGFP were obtained and grown in a GMO greenhouse and all had apparently normal morphology. The phenotypes of the 20 transgenic lines are shown in the Additional file 1: Figure S2.

### Timeline for transformation

It took about 32 weeks to obtain transformation results, starting from sowing seeds to PCR analysis on transformed seedlings right before transplanting to 1.7″ pots. However, the whole process could be reduced to 15 weeks with modifications. Firstly, always maintaining viable protocorms can save about 5 weeks, the amount of time needed for seeds to develop. Secondly, if seedlings are transferred to 1/2 MS not containing hygromycin; after the 3rd hygromycin selection, seedlings can grow rapidly. After 2 months of growth, a small piece of leaf tissue (1 × 1 mm) was excised for early PCR analysis. These modifications save time and increase efficiency of transformation.

### Frequency of transformation

Transformation rate is critical for the success of a transformation system. In order to know the transformation efficiency of this protocol, three batches of experiments were tested using Ubi:eGFP vector. As can be seen in Additional file 1: Table S1, transformation rate varied in different batches ranging from 1.2 to 5.2 %. Normally, healthy *Phal.* orchid capsule contain tens of thousands tiny seeds that appear as dust. A single capsule of *Phal. aphrodite* can be sown on 8–10 agar plates of 9 cm diameter. Each plate can germinate more than 1000 protocorms for *Agrobacteria* infection. Thus, the selection is rather easy and less labor intensive. Therefore, increase initial *Agrobacteria* infected protocorms plate numbers can apparently obtain more transgenic *Phal.* orchid lines.

### Molecular confirmation of transgenic lines

A total of 74 individual transgenic lines of *Phal.* orchids were obtained and planted in the greenhouse. Among them, 22 transgenic orchid pot seedlings (Additional file 1: Figure S2) were randomly selected, genomic DNA was isolated, and genomic PCR was performed. There was no *hygromycin phosphotransferase* gene (*HptII*) and enhanced green fluorescence protein (*eGFP*) amplification with the DNA from the Wt plant but obvious PCR banding was obtained in the positive control of the vector DNA (Fig. 2a). All of the 22 tested T0 transgenic lines showed *HptII* banding after the high stringency hygromycin selection (Fig. 2b). However, five T0 plants did not show *eGFP* bands (Fig. 2b). This might due to a problem with DNA insertion of eGFP in those lines. Overall, the T-DNA integration was accomplished normally in most of the transgenic lines.

To determine the T-DNA insertion number, Southern blot analysis was performed by randomly selecting some transgenic lines developed from the genetic background of Ama (2n) i.e., D18, D19 and D20; from Ama (4n) such as D1, and from L3 (4n) background such as D26, D28, and D32. Only the transgenic lines showed a hybridization signal, with no signal being seen in the Wt plants of Ama (2n), Ama (4n), and L3 (4n). Southern blot data indicated that most of the transgenic lines contained one or two copies of T-DNA insertion (Fig. 2c).

**Fig. 3:**
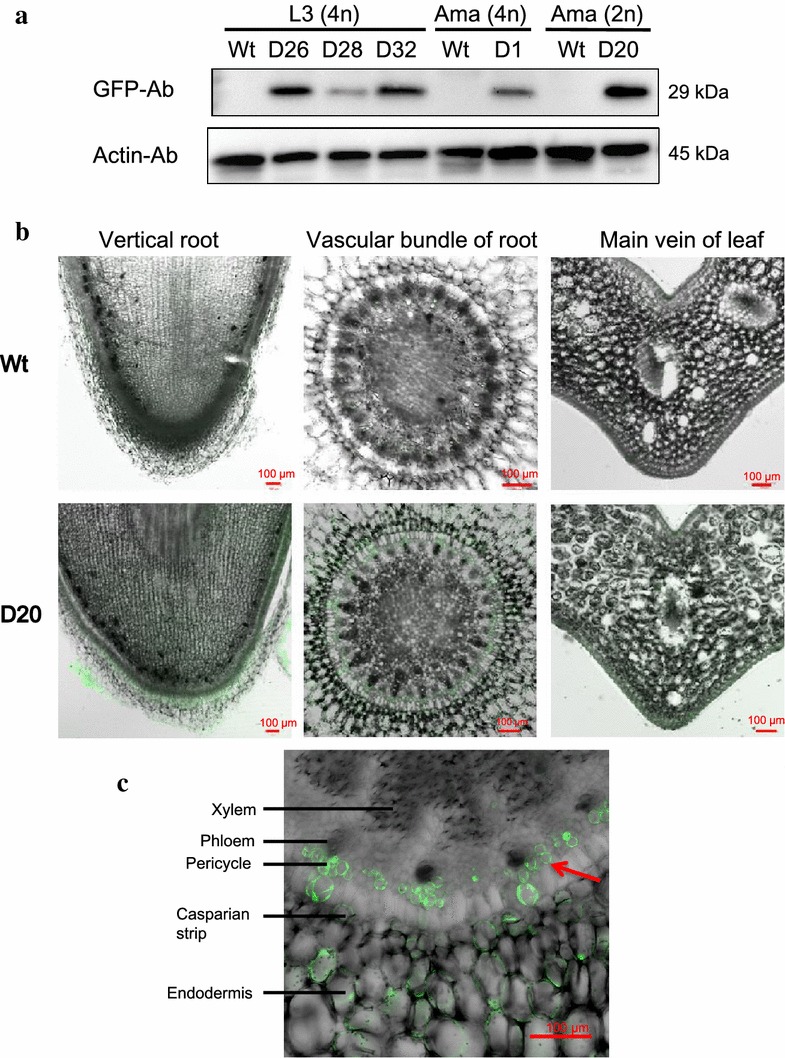
Western blot and detection of the GFP fluorescence signal in transgenic orchids. **a** Western blot analysis of the expression of GFP protein in transgenic orchids. Total protein was isolated from the leaf of orchid and 30 μg total protein was loaded into 10 % acrylamide gel, electrophoresed, and transferred onto polyvinylidene fluoride (PVDF) membrane for antibody probing. The membrane was probed using an anti-GFP antibody and anti-actin antibody served as the loading control. *Wt* wild-type; *4n* tetraploid; *2n* diploid. **b** GFP fluorescence signal was detected in D20 transgenic *Phalaenopsis aphrodite*. **c** A magnified view of the GFP signal in partial tissue of **b**. Root tissues of the D20 transgenic line and Wt were vertical and transversely sectioned, and leaves were transverse-sectioned using a vibratome and an image was obtained using a confocal microscope

Total protein was isolated from leaves of Ubi:eGFP transgenic orchid lines and their respective three wild types, L3 (4n), Ama (4n), and Ama (2n). Thirty micrograms of total protein was loaded and western blot analysis was performed using anti-eGFP antibody. Results indicated that none of the three wild types expressed the GFP signal but all the transgenic lines expressed eGFP protein in the T0 transgenic orchids (Fig. 3a). The lower panel showed anti-Actin antibody to ensure equal protein loading (Fig. 3a).

### Green florescent signal in orchid transgenic lines

Green florescent protein (GFP) is a marker for monitoring gene expression in transgenic plants (Hraska et al. 2006). Under confocal microscopy, as expected, there was an absence of GFP detection in the root and leaf of wild type but the GFP fluorescence signal was clearly observed in the D20 transgenic line, with more expression in the outer layers of the vertical root section (Fig. 3b). A strong GFP signal was seen in the cortex tissues in the transverse root and a very strong GFP signal was accumulated in the pericycle cells of the transgenic line as shown in Fig. 3c. The pericycle that was located between the endodermis and phloem is the tissue that enables plants to grow roots and facilitates plant development. Cells in the pericycle actively undergo cell division and proliferation (Dubrovsky et al. 2000). We observed more GFP signals around the pericycle under fluorescence microscope. Here we found the root pericycle close to the root tip has less endogenous GFP and it is good tissue to detect the expression of the GFP transgene (Fig. 3c).

### Uniform GUS expression in transgenic tissues

**Fig. 4:**
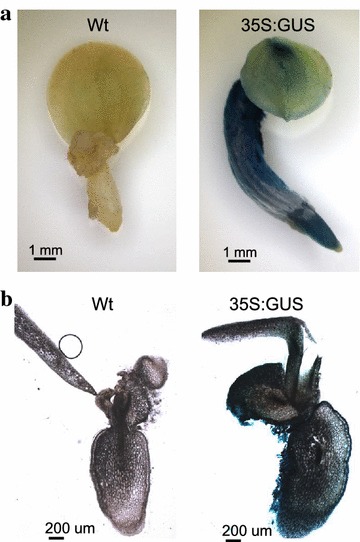
Histochemical Gus staining in transgenic *Phalaenopsis* orchid lines. Transgenic orchid explant harboring 35S:GUS and a Wt non-transgenic control were stained with X-Gluc and incubated at 37 °C overnight. **a** Image obtained using a Nikon SMZ1500 dissecting microscope (×7.5 magnification). **b** Explant tissue sections of approximately 120 µm in thickness were photographed on a Zeiss Axio Scope A1 microscope equipped with an Axio-Cam HRc camera (Zeiss, Germany) (×25 magnification)

Non-chimeric transgenic plants are essential for stable transformation. In order to check that the transformation protocol established in this study did not produce chimerism, T0 transgenic explants of 35S:GUS were stained with X-Gluc solution. All tissues showed strong GUS positive signals, including the leaf, root, and hypocotyl (Fig. 4a). Vibratome tissue section showed uniform GUS positive signals in all tissues and cells (Fig. 4b). These results confirmed that the transformation system established in this study did not produce chimeric transformants.

### Stable inheritance of transgene

**Fig. 5:**
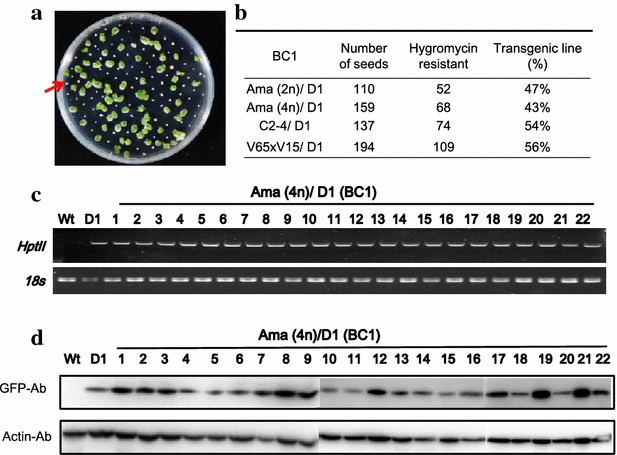
Stable inheritance of transgene in BC1 orchid progeny. **a** Pollinia of D1 transgenic line was crossed-pollinated to four distinct genetic backgrounds of Wt orchids and generated BC1 progenies. Ama (4n)/D1 BC1 seeds were sown on 1/2 MS agar plates containing 25 ppm hygromycin. Some BC1 progenies were resistant to hygromycin and some were susceptible showing white and wilt symptoms.* Red arrows* indicate hygromycin susceptible explants. **b** Record of survival rate in BC1 transgenic lines. **c** Genomic PCR showed *HptII* gene integration in the hygromycin-resistant BC1 progenies. **d** Western blot showed GFP protein expression in the hygromycin-resistant BC1 progenies. Ama (4n) was used as the non-transgenic wild-type negative control. D1, the original T0 transgenic parent was used as a positive control

It is very important to develop transgenic orchids with stable inheritance and consistent expression of target genes in the next generation. Therefore, pollinia of the D1 transgenic line were backcrossed to four distinct genetic backgrounds, Ama (4n, the original Wt of D1), Ama (2n), and the hybrids of C2-4 and V65 × V15, respectively, and produced BC1 seeds. Some BC1 progenies showed resistance in hygromycin selection (Fig. 5a). The hygromycin resistant explants varied from 43 to 56 % in four BC1 progenies (Fig. 5b). Southern blotting data indicated that D1 is a single T-DNA insertion transgenic line (Fig. 2c). BC1 progenies were heritable (Fig. 5b). Genomic PCR analysis showed that 22 hygromycin resistant BC1 of Ama (4n)/D1 seedlings all contained *HptII* amplicons, indicating that the introgression of transgenes is stable in the next generation of *Phal.* orchid (Fig. 5c). Moreover, western blot also indicated that all hygromycin survival progenies positively expressed eGFP protein (Fig. 5d). We concluded that the transgenic lines generated using the *Agrobacterium*-mediated young protocorm method presented in this study is stable and efficient. To be noted, the expression and introgression of transgenes to the next generation was demonstrated in this study.

## Discussion

*Phalaenopsis* orchids have been a mainstay of the global ornamental flower trade in recent years. Genetic engineering has emerged as a method with high potential to modify traits more precisely and enable functional genomics studies more efficiently. Although *Agrobacterium*-mediated transformation has been successfully applied to many agricultural or horticultural crops, it still remains difficult to apply to some plants that are not easily clonally propagated. Here, we reported an efficient method of *Agrobacterium*- mediated transformation using protocorms of *Phal. aphrodite*. Its advantages over previous methods can be summarized as follows:

We achieved a stable, efficient, yet less labor-intensive transformation method to genetically transform monocotyledonous *Phal.* orchids. Stable integration, expression, and inheritance of the transgenes were confirmed using molecular and genetic studies. To date, the transformation rates reported for *Phal.* orchids have been rather low and have never reached 2 % (Mishiba et al. 2005; Semiarti et al. 2007). However, this protocol could generate up to 5.2 % transformation efficiency (Additional file 1: Table S1).

Choose of appropriate size of target explants for transformation is very important for stable transformation. The orchid protocorms which are similar to immature embryos with extensive cell proliferation are good target for transformation. Chimerism could occur if large orchid embryos or PLBs are used as target tissues (Kuehnle and Sugii 1992). In the present study, we found small-size protocorms of 1.2 mm are suitable for transformation and we did not observe chimerism in transgenic explants after GUS staining (Fig. 4). Moreover, the inheritance of transgenes is stable in all the BC1 transformation lines tested in our genetic study as shown in Fig. 5. These data demonstrates that transformation protocol developed in this study is stable and heritable.

To speed up transformation process, we adopted three successive 25 ppm hygromycin selections at 2-week interval in this protocol. The total of 6 weeks in hygromycin selection media is much shorter than other transformation methods that usually take up to several months or longer. Selection under high concentration of hygromycin can reduce false positive transformants. In addition, using the Direct PCR Kit enables us to detect gene insertion in early stage to validate the positive transformants. These modifications significantly save time and increase efficiency of transformation. It is known that slow in growth is one of the bottlenecks for *Phal.* orchid production as well as for researches. Therefore, it is suggested once PCR confirms the transformants, immediately recover the transgenic seedlings in 1/2 MS agar without subjecting the pressure from antibiotics could speed up their growth dramatically.

It is known that GFP fluorescence has some weak points, such as the diminishing green fluorescence in older tissues, variation in fluorescence levels among different tissues, and interference with autofluorescence in plants (Hraska et al. 2006; Molinier et al. 2000). In the present study, we successfully detected the GFP fluorescent signal of transgenic *Phal.* orchid using a florescence microscope and enabled the determination of the presence of the transgene (Fig. 3b, c). Moreover, the transgene can be monitored non-destructively using a dissecting fluorescence microscope. The root tip can be partially cut to identify the GFP signal and will not affect the survival of the seedling. Interestingly, we found that pericycle cells close to root tip express high levels of GFP in this study (Fig. 3c). Higher cytoplasmic density in young tissues causing higher levels of expression of GFP than in older tissues has been previously described (Molinier et al. 2000). Therefore, it is suggested that the pericycle close to the root tip of orchid that contains dense proliferating cells could be a good tissue type to detect introgression of transgenes. We suggest that eGFP is a suitable reporter system that provides reliable results of introgression of transgenes and it is a good marker for the genetic transformation of *Phal.* orchids.

The *Agrobacterium*-mediated transformation system in this study may provide a practical tool for studying functional genomics, which can further assist the gene discovery and molecular breeding processes. GMO of ornamental crops that are not edible is more acceptable to consumers than GMO of food. Genome editing technology such as CRISPR (Liu et al. 2016; Zaidi et al. 2016; Zhu et al. 2016) provides the prospect of modification of orchids using highly efficient transformation techniques in *Phal.* orchids.

## Conclusions

This study established an *Agrobacterium*-mediated transformation system using orchid protocorm as a target explant. This protocol works well in tetraploid as well as in diploid *Phal. aphrodite* varieties. Moreover, this method is simple, stable, efficient, heritable, time saving, and less labor-intensive. In the era of big data and functional genomics, this transformation method can significantly contribute to the gene discoveries, genetic engineering and general R&D of *Phal.* orchid.

## Additional file

**Additional file 1: Table S1.** Transformation efficiency in different batches of experiments. TH274 × TH601 hybrid seeds were sown in 1/2 MS containing 25 ppm hygromycin and the surviving protocorms that were recorded from the first to the third round of selections. **Figure S1.** *Phalaenopsis* orchid cultivars used for transformation in this study. a Plant type of *Phalaenopsis* orchid cultivars at flowering stage. b Flower morphology of *Phalaenopsis* orchid cultivars. 4n, tetraploid; 2n, diploid. Bars = 5 cm (a), 2 cm (b). **Figure S2.** Phenotype of T0 transgenic orchid seedlings overexpressing Ubi:GFP. D1 to D33 represented the individual T0 transgenic lines overexpressing Ubi:GFP. The respectively wild type is written in the right hand side.
