# Supplementary material for: Efficient and heritable transformation of Phalaenopsis orchids
Source: Bot Stud. 2016 Oct 20;57:30. doi: 10.1186/s40529-016-0146-6 (PMC5430590; doi:10.1186/s40529-016-0146-6)
Supplement: Supplementary file 1 — Additional file 1: Table S1. Transformation efficiency in different batches of experiments. TH274 × TH601 hybrid seeds were sown in 1/2 MS containing 25 ppm hygromycin and the surviving protocorms that were recorded from the first to the third round of selections. Figure S1. Phalaenopsis orchid cultivars used for transformation in this study. a Plant type of Phalaenopsis orchid cultivars at flowering stage. b Flower morphology of Phalaenopsis orchid cultivars. 4n, tetraploid; 2n, diploid. Bars = 5 cm (a), 2 cm (b). Figure S2. Phenotype of T0 transgenic orchid seedlings overexpressing Ubi:GFP. D1 to D33 represented the individual T0 transgenic lines overexpressing Ubi:GFP. The respectively wild type is written in the right hand side. [file 40529_2016_146_MOESM1_ESM.pdf]

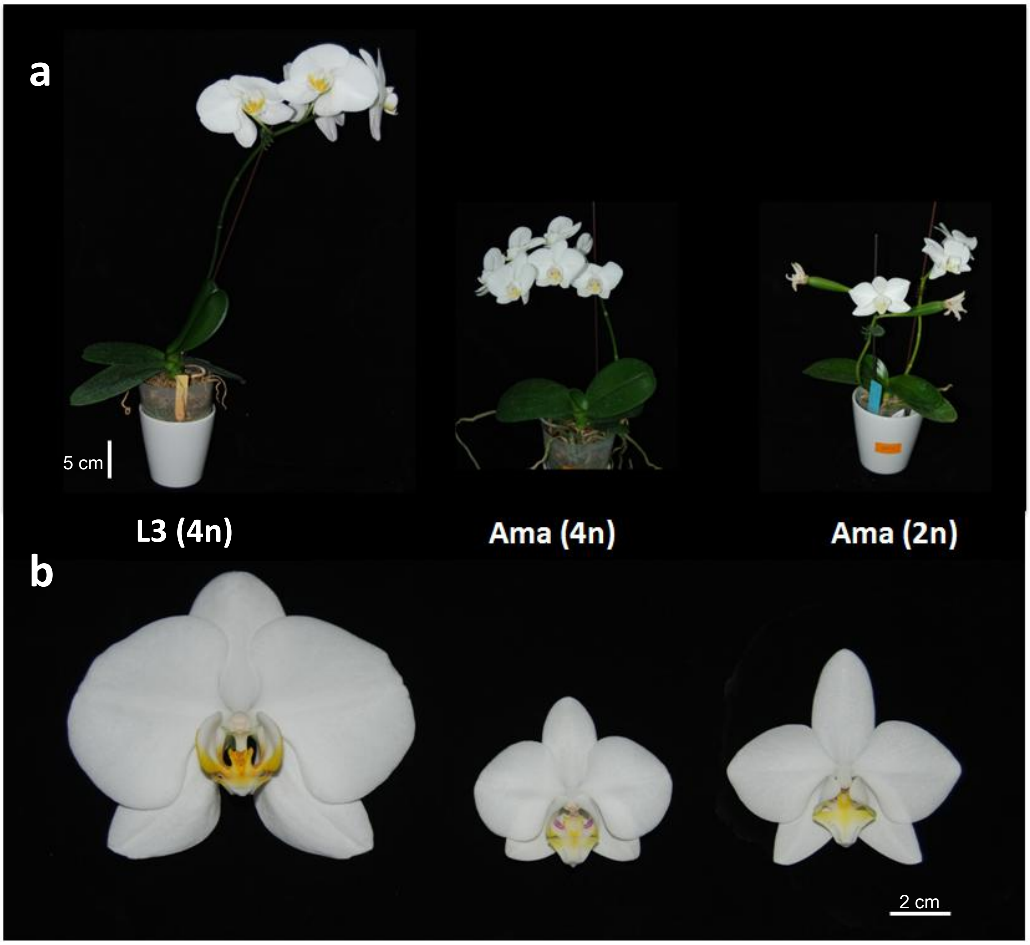

Fig. S1

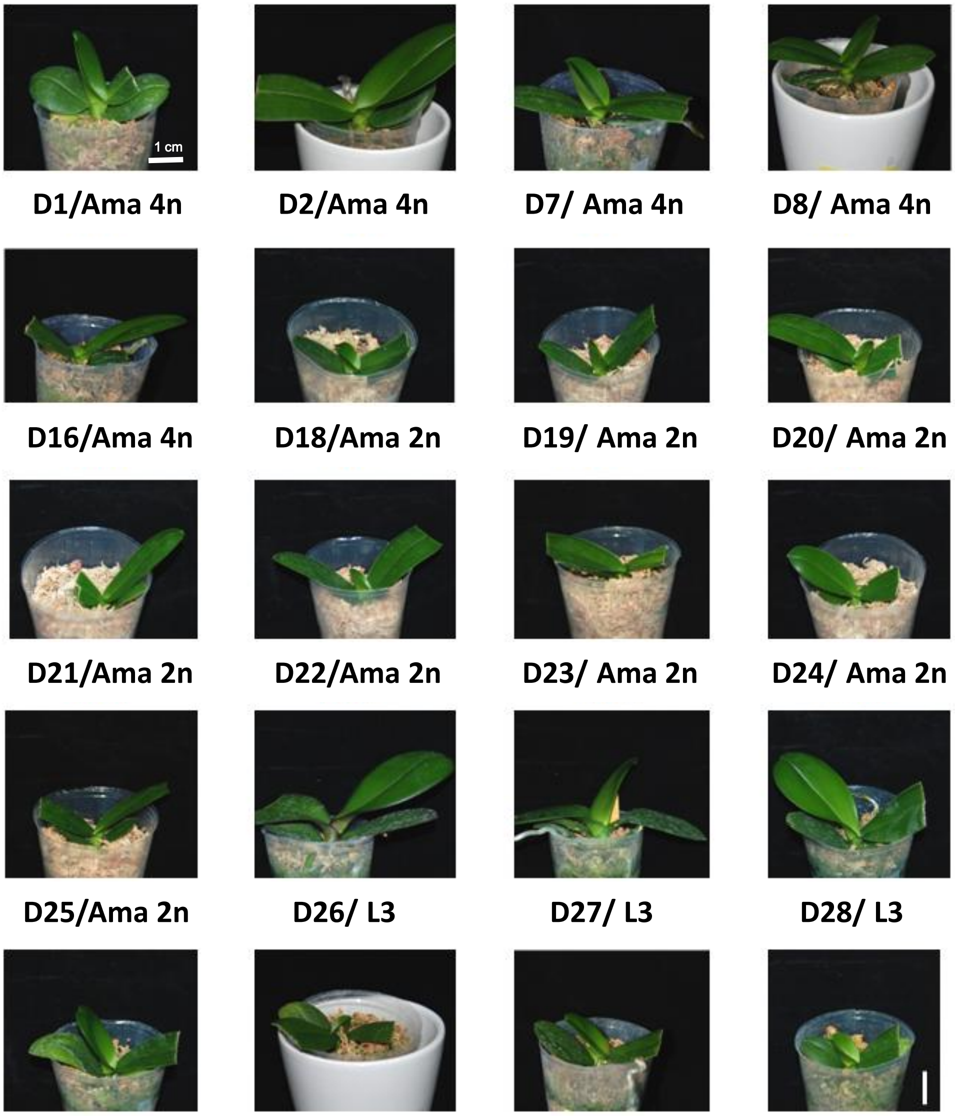
1 cm
D1/Ama 4n
D2/Ama 4n
D7/ Ama 4n
D8/ Ama 4n
D16/Ama 4n
D18/Ama 2n
D19/ Ama 2n
D20/ Ama 2n
D21/Ama 2n
D22/Ama 2n
D23/ Ama 2n
D24/ Ama 2n
D25/Ama 2n
D26/ L3
D27/ L3
D28/ L3
D30/ L3
D31/ L3
D32/ L3
D33/ L3

Fig. S2

Table S1 Transformation efficiency in different batches of experiments.

Experiment	Total protocorm	Hygromycin resistance explant No.			Transformation rate %
		1st selection	2nd selection	3rd selection	
Experiment-1	3263	1271	659	171	5.2
Experiment-2	1452	356	43	19	1.3
Experiment-3	3294	753	112	40	1.2
**Average**					**2.6**
